# Genetic control of flowering time and fruit yield in citron watermelon

**DOI:** 10.3389/fpls.2023.1236576

**Published:** 2023-10-10

**Authors:** Dennis N. Katuuramu, Amnon Levi, William P. Wechter

**Affiliations:** U.S. Vegetable Laboratory, Agricultural Research Service, U.S. Department of Agriculture, Charleston, SC, United States

**Keywords:** watermelon, flowering time, fruit yield components, genome-wide association analysis, marker-trait association

## Abstract

Flowering time and fruit yield are important traits in watermelon crop improvement. There is limited information on the inheritance and genomic loci underlying flowering time and yield performance, especially in citron watermelon. A total of 125 citron watermelon accessions were evaluated in field trials over two growing seasons for days to male and female flowers, fruit count, fruit weight, and fruit yield. The germplasm was genotyped with more than two million single-nucleotide polymorphism (SNP) markers generated via whole-genome resequencing. Trait mapping was conducted using a genome-wide association study (GWAS). Broad-sense heritability for all traits ranged from moderate to high, indicating that genetic improvement through breeding and selection is feasible. Significant marker-trait associations were uncovered for days to female flower (chromosomes Ca04, Ca05, Ca08, and Ca09), fruit count (on Ca02, Ca03, and Ca05), fruit weight (on Ca02, Ca06, Ca08, Ca10, and Ca11), and fruit yield on chromosomes Ca05, Ca07, and Ca09. The phenotypic variation explained by the significant SNPs ranged from 1.6 to 25.4, highlighting the complex genetic architecture of the evaluated traits. Candidate genes relevant to flowering time and fruit yield component traits were uncovered on chromosomes Ca02, Ca04, Ca05, Ca06, Ca09, and Ca11. These results lay a foundation for marker-assisted trait introgression of flowering time and fruit yield component traits in watermelons.

## Introduction

Sweet-fleshed watermelon (*Citrullus lanatus*) is an economically important vegetable crop grown and consumed worldwide (FAOSTAT, 2022). The fruit is rich in moisture, sugars, and bioactive compounds (Maoto et al., 2019; Zamuz et al., 2021). Due to centuries of domestication and improvement sweeps, sweet-fleshed watermelon underwent a significant genetic bottleneck and exhibits low genetic diversity (Levi et al., 2001). Conversely, *Citrullus amarus* (citron watermelon) displays significant genetic variation for traits important in the breeding and improvement of sweet-fleshed watermelons (Levi et al., 2013). Both *C. lanatus* and *C. amarus* belong to the Cucurbitaceae family of fruit vegetable crops with significant global economic importance (Wehner, 2008). *C. lanatus* and *C. amarus* have comparable genome sizes of 425 Mb and 423.2 Mb, respectively (Guo et al., 2013; Wu et al., 2023). Close relatives to *C. lanatus* such as *C. amarus* have been used in controlled crosses to expand the genetic base for cultivated watermelon improvement (Wehner, 2008; Jarret et al., 2017). Continuous evaluation and genetic mapping of native traits within *C. amarus* germplasm will be helpful in the design of introgression and improvement strategies for successful watermelon cultivar development.

Flowering time is a crucial fitness trait that has influenced domestication and the extent of dissemination to new climatic regions for many crop species (Blumel et al., 2015). Flowering time determines the crop’s reproductive success and breeding methodology that can be used for cultivar development (Jung and Muller, 2009). Watermelon displays several flower sex expression patterns including monoecy (unisexual male and female flowers develop on the same plant) and andromonoecy [staminate and perfect flowers are present on the same plant] (Bhowmick and Jha, 2015; Ji et al., 2015). Male flowers open first followed by female flowers at a ratio of approximately 7:1 (Wehner, 2008). The dominant sex expression system is monoecy, and it is the preferred form for commercial breeding of inbred and hybrid watermelon genotypes (Grumet and Taft, 2012; Ji et al., 2015). Flowering time in watermelon is a polygenically inherited trait with large genomic regions identified on chromosomes 2, 3, and 11 in *C. lanatus* biparental crosses (McGregor et al., 2014; Gimode et al., 2020). In melon (*Cucumis melo*), polygenic associations for flowering time have been reported on chromosomes 6 and 7 (Perpina et al., 2016). In cucumbers, QTLs for flowering time have been detected on chromosomes 1 and 6 (Sheng et al., 2020). Phenological traits like flowering time and days to maturity can influence fruit yield performance in watermelon. Understanding the genetic bases of flowering time can inform crop breeding strategies, thus contributing to the prediction of yield risks such as disease outbreaks, heat, and drought stresses.

Improving fruit yield is one of the major targets for both public and private watermelon breeding programs (Mohr, 1986; Gusmini and Wehner, 2005). Fruit yield, fruit count, and average fruit weight are some of the key drivers of the marketability and profitability of the watermelon produce industry (Kaiser, 2012). Fruit yield is a polygenic trait with low-to- moderate heritability and is the compound of multiple interacting component traits such as vine architecture, number of plants per area (plant density), fruit count per plant, and fruit weight (Wehner, 2008; Kumar and Wehner, 2013). All these yield component traits are quantitative in nature and are based on the interaction of physiological and morphological characteristics of the plant as well as environmental factors (Kumar and Wehner, 2011; Dia et al., 2016). There have been repeated and long-term selection efforts for acceptable consumer fruit quality and biotic stress tolerance traits in watermelon and other cucurbit crops (Levi et al., 2001). However, there have been limited efforts to genetically map flowering time and fruit yield component traits in watermelon. Understanding the genetic architecture of fruit yield and its interaction with the individual yield components offers a basis for genetic improvement in watermelon. Additionally, the identification of genomic regions controlling fruit yield component traits is essential for marker-assisted selection, which ultimately enhances genetic gains for yield in watermelon breeding.

Advances in next-generation sequencing technologies have enabled the generation of chromosome-scale genomes and high-density single-nucleotide polymorphism (SNP) molecular marker datasets in watermelon (Guo et al., 2019; Wu et al., 2019; Katuuramu et al., 2022). These genome sequence resources provide new capabilities to characterize genetic diversity and map loci underlying the phenotypic expression of important traits in watermelons. Mapping strategies such as genome-wide association study (GWAS) involve the assembly of germplasm diversity panels followed by genotyping and phenotyping (Zhu et al., 2008; Ingvarsson and Street, 2011). The GWAS approach relies on high-density SNP markers, historical meiotic events, and reliable phenotypic data to uncover marker-trait associations (Zhu et al., 2008; Ingvarsson and Street, 2011). Identification of genomic regions that influence flowering time and fruit yield will be useful during marker-assisted trait introgressions. The overall objective of this research was to understand the genetic architecture of phenological and fruit yield component traits in citron watermelon. The specific objectives were i) to evaluate the variation in phenological and fruit yield component traits present in citron watermelon collection and ii), using GWAS, to analyze genomic regions (significant SNPs and candidate genes) underlying the observed phenotypic variation. In this research, a collection of 125 citron watermelon germplasm accessions was evaluated under field conditions for flowering time and fruit yield component traits. The GWAS procedure was deployed to uncover marker-trait associations using SNP markers generated from whole-genome resequencing.

## Materials and methods

### Plant germplasm and transplant production

A total of 125 citron watermelon accessions were obtained from the United States Department of Agriculture (USDA) germplasm repository in Griffin, GA, USA (**Supplementary Table S1**). Individual accessions were maintained through regular cycles of controlled self-pollinations to reduce heterozygosity and enhance germination ability. The majority of the citron watermelon accessions (112) were collected/received from Africa, three were collected/received from Asia, five were collected from Europe, and five were received from North America (**Supplementary Table S1**). Seeds for each accession were sown directly into Metro-Mix 360 soilless media (Sun Gro Horticulture, Agawam, MA, USA) in 36-cell vegetable propagation trays (120-cm^3^ cell size; T.O. Plastics Inc., Clearwater, MN, USA). To ensure optimum growth, citron watermelon seedlings were watered as needed, and a balanced water-soluble (NPK: 20:20:20) fertilizer was applied at a rate of 5 g/L (Scotts, Marysville, OH, USA). Greenhouse temperatures ranged from 18°C to 38°C with an average of 25°C. Seedlings were grown in the greenhouse for 4 weeks before being transplanted to the field plots.

### Field experiments

Field studies were conducted at the United States Vegetable Laboratory research station in Charleston, South Carolina. The soils at the research site are predominantly Yonges loamy fine sand with a pH of 6.0, organic matter of 0.9%, and cation exchange capacity of 4.7 meq/100 g. Site preparation, seeding, transplanting, irrigation, fertigation, and pest and disease management were similar in both the 2019 and 2022 field seasons as described below. Prior to making field beds, the research site was cultivated twice, first using a tractor-mounted offset disc harrow (John Deere, Model 425) and second with the tractor-mounted Perfecta field cultivator (Unverferth, Kalida, OH, USA). The two tillage methods were used in order to cut, loosen, and smoothen the soil as well as destroy any winter/spring vegetation before forming raised beds and laying the plastic mulch. The raised field beds were made using a Kennco Superbedder (Kennco Manufacturing Co., Ruskin, FL, USA). The field beds were 121.9 m long, 91.4 cm wide, and 20.3 cm high. The distance between beds was 3.4 m. The beds were sprayed with herbicides and the next day covered with a black and white totally impermeable film (TIF) plastic mulch (0.03 mm thick; Polygro LLC, Safety Harbor, FL, USA) using a Kennco Superbedder prior to transplanting.

The day before transplanting, a tractor-mounted hole puncher was used to make planting holes (approx. 7.6 cm deep and 5.1 cm in diameter) for the watermelon seedlings. In both years, there were three plants per genotype replicated three times. Within the genotype, spacing was 1.8 m, while the space between genotypes was 2.7 m. Four-week-old watermelon seedlings were transplanted by hand to the research fields on May 16, 2019, and April 27, 2022. The experimental design in both the 2019 and 2022 field seasons was a randomized complete block design with three replications.

To ensure optimum plant growth, sub-surface drip irrigation was applied using drip tapes placed centrally underneath the plastic mulch on all raised beds prior to transplanting. The drip tape specifications were 16-mm hose diameter, 0.20-mm wall thickness, and 30-cm emitter spacing, with an emitter flow rate of 1.0 L/h when the pressure regulator was set to 8 psi (Aqua-Traxx^®^, Toro Agricultural Irrigation, El Cajon, CA, USA). In both the 2019 and 2022 field seasons, Possum’s 10-0-10 (N-P-K) PLUS fertilizer was utilized to ensure optimal plant growth (Possum’s West, Charleston, SC, USA). Fertilization was conducted via injection into the drip irrigation system with approximately 168 kg/ha of nitrogen applied over the entire growing season. Application of phosphorus fertilizer was not necessary for the fertigation program since soils at the research fields had high natural levels (> 210 kg/ha) of this nutrient based on the soil test results at the time of transplanting in both field seasons.

Biological pests in the research plots were controlled by physical and chemical means. Weeds were managed using a black and white TIF plastic mulch (Polygro LLC, Safety Harbor, FL, USA) and a single application before transplanting of broadleaf herbicides: Sandea^®^ (Gowan), Dual Magnum^®^ (Syngenta), and Prowl H2O^®^ (BASF). During the growing seasons, additional weeds were pulled by hand on a weekly basis. Fungicides to control foliar disease outbreaks during the two growing seasons were applied weekly, and in rotation, they included Inspire Super^®^ (Syngenta), Quintec^®^ (Gowan), Proline^®^ (Bayer), and Initiate 720^®^ (Loveland Products). Weather data (precipitation, minimum and maximum temperatures, and relative humidity) during the two growing seasons were retrieved from the Southeast Regional Climate Center (https://sercc.com accessed on February 27, 2023).

### Phenotypic data collection

A total of five adaptive and yield component traits were evaluated in this research. Days to male and female flowers was recorded as the number of days from seeding to when the two flower types were first observed while walking through the genotype plots. Within every genotype, fruits were declared to be physiologically ripe and mature (ready for harvest) upon detection of a brown and dry tendril at the node bearing the fruit, as well as a dull waxy fruit surface and light-colored ground spot on the fruit (Maynard, 2001). Yield component traits included fruit count, fruit weight, and fruit yield. Fruit count was recorded as the average number of fruits per plant. Fruit weight was recorded as the average weight per fruit and recorded in kg. Fruit yield was recorded as the weight of fruits per genotype plot and converted to kg/ha. The majority of the citron watermelon genotypes exhibited a concentrated (uniform) fruit-set pattern. For accessions with indeterminate (non-uniform) fruit sets, fruit yield component trait (count, weight, and yield) data were derived from the sum of values from multiple cuts following the final harvest.

### Statistical analysis

Analysis of variance was performed using the MIXED procedure in SAS v9.4 (SAS Institute, 2013) according to this model: Trait = µ + Genotype + Year + Genotype-by-Year + rep(Year) + Error. Pearson’s correlation analysis among traits was conducted using the CORR procedure in SAS v9.4 (SAS Institute, 2013). Variance components for computing broad-sense heritability (H^2^) were generated using the VARCOMP procedure in SAS v9.4 using restricted maximum likelihood estimation (REML) with all effects in the model treated as random (SAS Institute, 2013). The broad-sense heritability on an entry-mean basis was calculated following the formula provided by Holland et al., 2003. Trait data were converted to best linear unbiased estimates (BLUEs) in SAS v9.4, which were then used to conduct GWAS.

### SNP genotyping and genome-wide association analysis

Details about DNA isolation and whole-genome resequencing can be found in Katuuramu et al. (2022). Briefly, whole-genome resequencing data for the 125 citron watermelon (*C. amarus*) accessions were obtained using Illumina NovaSeq 6000 Sequencing Technology (Illumina, San Diego, CA, USA). Shotgun genomic libraries were sequenced on a single lane using the Illumina NovaSeq 6000 machine to generate 150-bp paired-end reads. Reads were filtered and trimmed as presented previously in Katuuramu et al. (2022). Variant discovery and quality control were conducted using the GATK v3.6 best practices workflow (McKenna et al., 2010; Depristo et al., 2011; van der Auwera et al., 2013). After filtering for minor allele frequency (MAF < 0.05) and missing data > 10%, a total of 2,126,759 SNPs were available for downstream analyses. Population structure and linkage disequilibrium patterns present in this *C. amarus* collection have been reported previously by Katuuramu et al. (2022). Kinship was examined using the VanRaden method implemented in GAPIT v3.0 within R v4.2.0 (VanRaden, 2008; Lipka et al., 2012; Wang and Zhang, 2021; R Core Team, 2022). Several single- and multi-locus models were evaluated for their suitability to perform GWAS on the trait data generated in this research. Results from fixed and random model circulating probability unification (FarmCPU) and Bayesian-information and linkage-disequilibrium iteratively nested keyway (BLINK) showed better model fitting of the data based on the quantile–quantile (QQ) plots. Both the BLINK and FarmCPU are multi-locus GWAS models, have increased statistical power, can handle large marker datasets, can better control false positives and negatives, and are computationally more efficient when compared to the preceding single-locus models like general and mixed linear models [GLM or MLM] (Liu et al., 2016; Huang et al., 2018). Principal components (PCs) to account for population stratification during GWAS were generated using the *prcomp* function in R v4.2.0 (R Core Team, 2022). The optimum number of PCs to include as covariates for every trait was determined using scree plot analysis and Bayesian information criterion-based selection procedure in GAPIT v3.0 (Schwarz, 1978; Lipka et al., 2012; Wang and Zhang, 2021; R Core Team, 2022). Manhattan and QQ plots were generated in R v4.2.0 using the *CMplot* package (R Core Team, 2022; Yin, 2022). Significant marker-trait associations were established using the false discovery rate (FDR) method at an alpha level of 0.05 (Benjamini and Hochberg, 1995). The likelihood ratio-based *R* ^2^ statistic was used to compute the phenotypic variation explained (PVE) by the significant SNP markers for every trait (Sun et al., 2010). Comparison of the allelic effects of the significant SNP markers on the phenotypic means was performed using Student’s two-tailed *t*-test and visualized in R v4.2.0 (R Core Team, 2022). Candidate genes were selected within a ±85-kb window from the significant SNP for the traits of interest using the annotated USVL 246-FR2 *C. amarus* reference genome (http://cucurbitgenomics.org/). The search interval was chosen based on the linkage disequilibrium pattern present in this citron watermelon collection (Katuuramu et al., 2022). Putative candidate genes were those whose annotation, description, and gene ontology functions are relevant to molecular control of the traits evaluated in this research.

## Results

### Weather data

**Figure 1:**
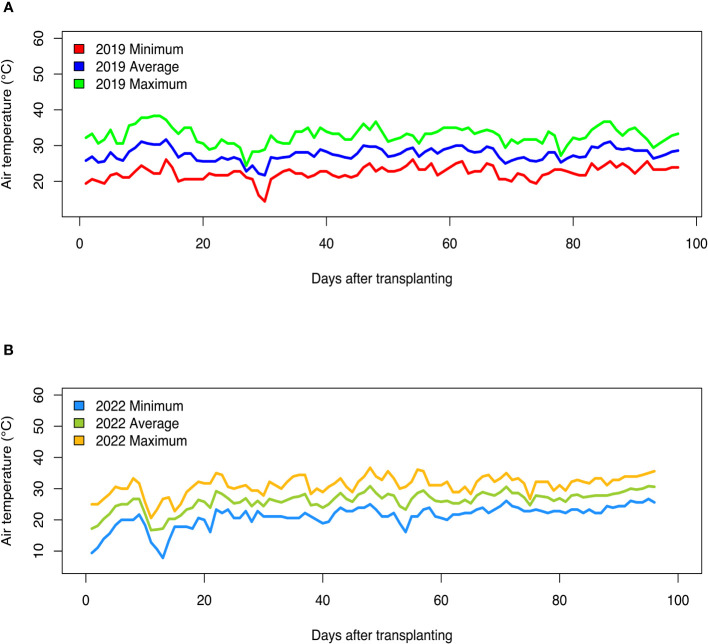
Daily air temperature (minimum, average, and maximum) recorded during the **(A)** 2019 and **(B)** 2022 field growing seasons at the U.S. Vegetable Laboratory research station in Charleston, South Carolina.

**Figure 2:**
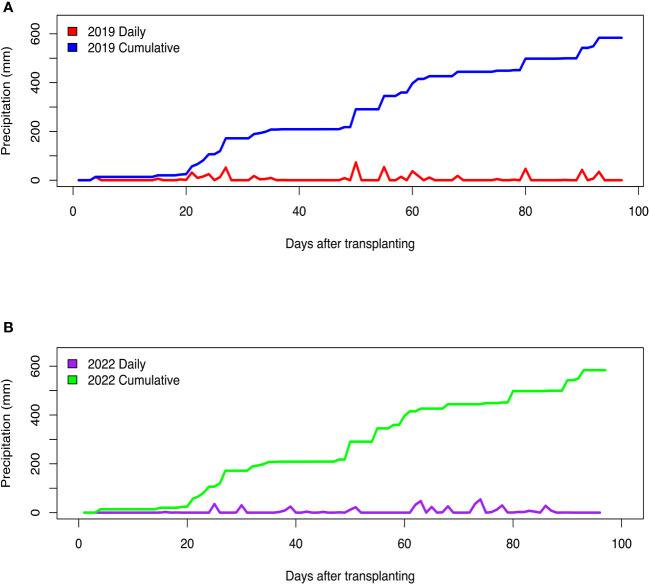
Daily and cumulative precipitation received during the **(A)** 2019 and **(B)** 2022 field growing seasons at the U.S. Vegetable Laboratory research station in Charleston, South Carolina.

Fluctuations in air temperature and precipitation were observed during the two field seasons (**Figures 1**, **2**). In the 2019 growing season, daily minimum air temperature ranged from 14.4°C to 26.1°C, while daily maximum air temperature readings varied from 24.4°C to 38.3°C (**Figure 1A**). In the 2022 field season, daily minimum air temperature ranged from 7.8°C to 26.7°C, while daily maximum temperature readings varied from 20.6°C to 36.7°C (**Figure 1B**). In the 2019 growing season, the total precipitation received was 583.7 mm, while in the 2022 season, the total rainfall received was 469.4 mm (**Figure 2**). Although total precipitation was higher in the 2019 growing season, there was a period of 64 days without rainfall (**Figure 2A**). During the 2022 growing season, there was a period of 61 days without rainfall (**Figure 2B**). The average daily relative humidity (RH) ranged from 54.1% to 94.2% in the 2019 growing season, while during the 2022 field season, RH varied from 47.7% to 91.4%.

### Phenotypic variation, heritability, and trait correlations

**Figure 3:**
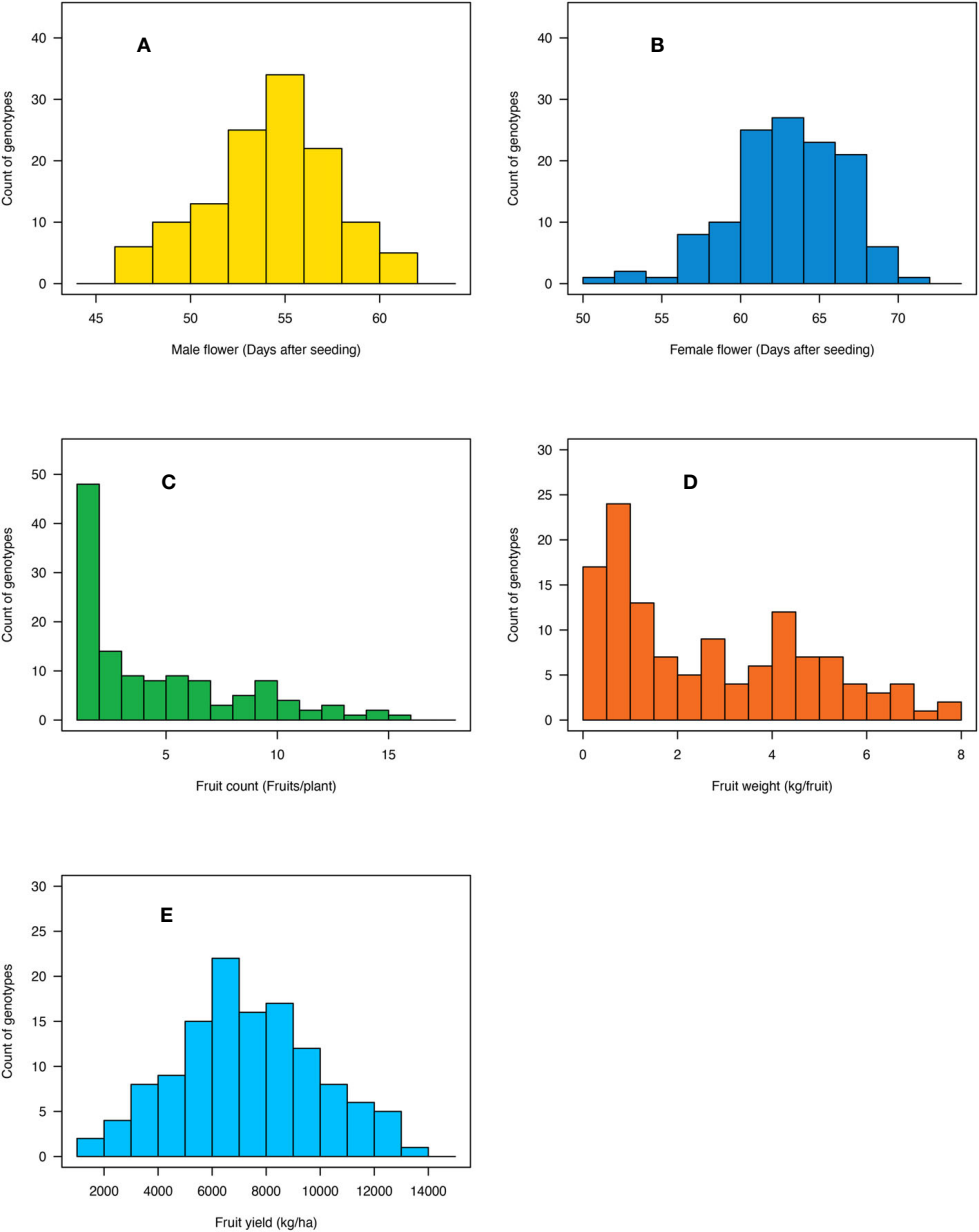
Histogram showing the distribution of **(A)** days to male flower, **(B)** days to female flower, **(C)** fruit count, **(D)** fruit weight, and **(E)** fruit yield across the 125 citron watermelon genotypes averaged over the two field growing seasons at the U.S. Vegetable Laboratory research station in Charleston, South Carolina.

Days to male flower ranged from 47 to 62 days post-seeding with a fold variation of 1.3 (**Table 1**; **Figure 3A**). Days to female flower ranged from 52 to 71 days after seeding with a fold variation of 1.4 (**Table 1**; **Figure 3B**). Both days to male and female flower traits had a moderately high heritability of 0.75 (**Table 1**). Fruit count ranged from one to 16 fruits per plant and had a heritability of 0.89 (**Table 1**; **Figure 3C**). Fruit weight ranged from 0.2 to 7.6 kg per fruit and had a fold variation of 38 and a heritability of 0.84 (**Table 1**; **Figure 3D**). Fruit yield ranged from 1,331 to 13,178 kg/ha and had a fold variation of 9.9 and a moderate heritability of 0.65 (**Table 1**; **Figure 3E**). Days to male flower was positively correlated with days to female flower (*r* = 0.72) and fruit weight (*r* = 0.29) but negatively correlated with fruit count (*r* = −0.37) (**Table 2**). Days to female flower was negatively correlated to fruit count (*r* = −0.46) but positively correlated to fruit weight (*r* = 0.39) (**Table 2**). Fruit count was negatively correlated to both fruit weight (*r* = −0.77) and fruit yield (*r* = −0.41) (**Table 2**). Fruit weight was positively correlated with fruit yield (*r* = 0.71) (**Table 2**).

**Table 1:** Summary statistics for flowering time and fruit yield component traits across the 125 citron watermelon genotypes evaluated over two field growing seasons at the U.S. Vegetable Laboratory research station in Charleston, South Carolina.

Traits	Mean ± SD	Min	Max	Fold variation	H^2^
Male flower (days after seeding)	55 ± 3	47	62	1.3	0.75
Female flower (days after seeding)	63 ± 4	52	71	1.4	0.75
Fruit count (fruits/plant)	5 ± 4	1	16	16	0.89
Fruit weight (kg/fruit)	2.7 ± 2.1	0.2	7.6	38	0.84
Fruit yield (kg/ha)	7,312 ± 2,605	1,331	13,178	9.9	0.65

**Table 2:** Correlation among flowering time and fruit yield component traits across the 125 citron watermelon genotypes evaluated over two field growing seasons at the U.S. Vegetable Laboratory research station in Charleston, South Carolina.

Traits	Male flower	Female flower	Fruit count	Fruit weight	Fruit yield
Male flower	–	0.72**	−0.37**	0.29*	−0.002 ns
Female flower		–	−0.46**	0.39**	−0.01 ns
Fruit count			–	−0.77**	−0.41**
Fruit weight				–	0.71**
Fruit yield					–

ns, not significant.

*Significant at the 0.001 probability level.

**Significant at the 0.0001 probability level.

### Marker-trait associations and candidate genes

**Figure 4:**
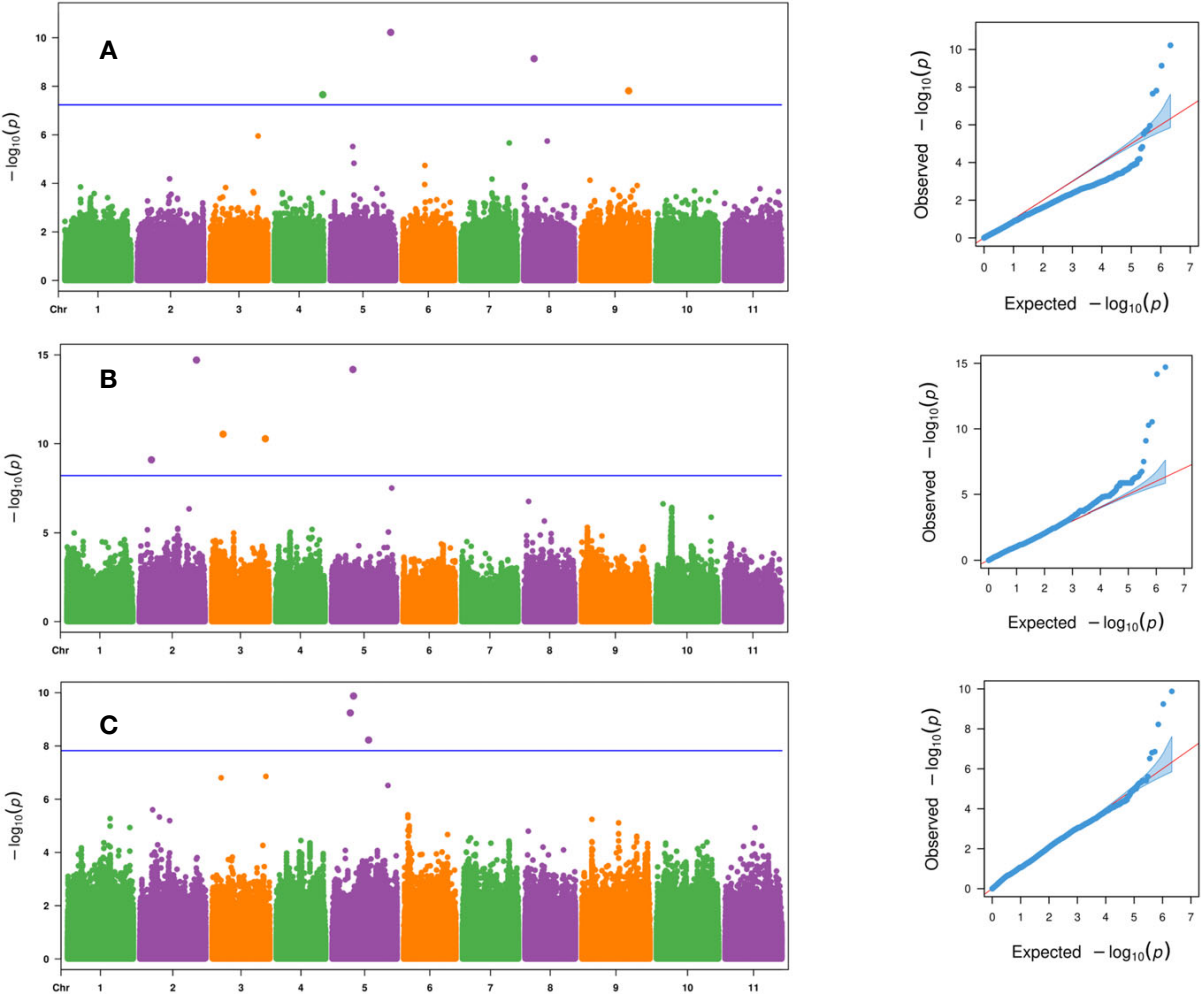
Manhattan and QQ plots for the evaluated traits of **(A)** days to female flower based on the FarmCPU model, **(B)** fruit count based on the FarmCPU model, and **(C)** fruit count based on the BLINK model across the 125 citron watermelon genotypes using BLUEs from the two field growing seasons. The blue solid horizontal line is an FDR cutoff of α = 0.05. QQ, quantile–quantile; BLUEs, best linear unbiased estimates; FDR, false discovery rate.

Four SNP markers were significantly associated with days to female flower (*p*- value ≤ 2.2 × 10^−8^) and were located on chromosomes Ca04, Ca05, Ca08, and Ca09 (**Table 3** and **Figure 4A**). These four significant SNPs were all uncovered by the FarmCPU GWAS model and explained from 2% to 25.1% of the phenotypic variation in days to female flower (**Table 3** and **Figure 4A**). There were seven significant SNPs associated with fruit count (*p*- value ≤ 8.1 × 10^−10^) and were distributed on chromosomes Ca02, Ca03, and Ca05 (**Table 3**; **Figures 4B, C**). Two SNP markers were located on chromosome Ca02 (S2_6939423 and S2_32688327), while two SNPs were located on chromosome Ca03 (S3_6555036 and S3_30701734). Three SNP markers were located on chromosome Ca05, which included S5_10364285, S5_12220057, and S5_20822179 (**Table 3**; **Figures 4B, C**). Four of the significant SNPs were unique to the FarmCPU GWAS model results, while two were found only with the BLINK model (**Table 3**; **Figures 4B, C**). The second most significant SNP marker (*p*- value = 6.6 × 10^−15^) was located on chromosome Ca05 and was uncovered by both the BLINK and FarmCPU models (**Table 3**). The phenotypic variation in fruit count explained by these seven significant SNP markers ranged from 2.9% to 13.5% (**Table 3**).

**Table 3:** Details of significant SNPs associated with flowering time and fruit yield component traits across the 125 citron watermelon genotypes evaluated over two field seasons at the U.S. Vegetable Laboratory research station in Charleston, South Carolina.

Traits	Chr.	SNP	SNP position (bp)	SNP *p*- value	MAF	Major allele	Minor allele	PVE (%)	GWAS model
Days to female flower	Ca04	S4_27578802	27,578,802	2.2E−08	0.44	G	A	2.0	FarmCPU
	Ca05	S5_34377129	34,377,129	6.1E−11	0.06	T	A	25.1	FarmCPU
	Ca08	S8_5886042	5,886,042	7.3E−10	0.12	G	A	11.7	FarmCPU
	Ca09	S9_27213639	27,213,639	1.5E−08	0.09	G	A	12.3	FarmCPU
Fruit count	Ca02	S2_6939423	6,939,423	8.1E−10	0.19	G	A	3.9	FarmCPU
	Ca02	S2_32688327	32,688,327	1.9E−15	0.11	A	C	7.1	FarmCPU
	Ca03	S3_6555036	6,555,036	2.9E−11	0.08	C	T	5.0	FarmCPU
	Ca03	S3_30701734	30,701,734	5.3E−11	0.21	C	T	2.9	FarmCPU
	Ca05	S5_10364285	10,364,285	5.7E−10	0.10	G	A	8.7	BLINK
	Ca05	S5_12220057	12,220,057	6.6E−15	0.05	G	A	13.5	BLINK, FarmCPU
	Ca05	S5_20822179	20,822,179	5.9E−09	0.08	C	T	10.0	BLINK
Fruit weight	Ca02	S2_34729922	34,729,922	2.3E−09	0.07	C	G	9.2	FarmCPU
	Ca06	S6_14558842	14,558,842	2.5E−14	0.27	T	C	25.4	BLINK, FarmCPU
	Ca08	S8_12372627	12,372,627	4.4E−13	0.39	G	A	13.1	FarmCPU
	Ca10	S10_14635976	14,635,976	9.6E−10	0.37	A	G	1.6	FarmCPU
	Ca11	S11_3991224	3,991,224	1.4E−10	0.44	G	A	8.4	FarmCPU
	Ca11	S11_20119275	20,119,275	1.8E−10	0.12	T	C	7.4	BLINK
Fruit yield	Ca05	S5_6875362	6,875,362	2.6E−09	0.46	T	G	17.2	FarmCPU
	Ca07	S7_30735801	30,735,801	1.3E−10	0.42	G	A	21.6	FarmCPU
	Ca09	S9_17168406	17,168,406	5.7E−11	0.08	G	T	11.4	FarmCPU

SNPs, single-nucleotide polymorphisms; MAF, minor allele frequency; PVE, phenotypic variation explained; GWAS, genome-wide association study.

**Figure 5:**
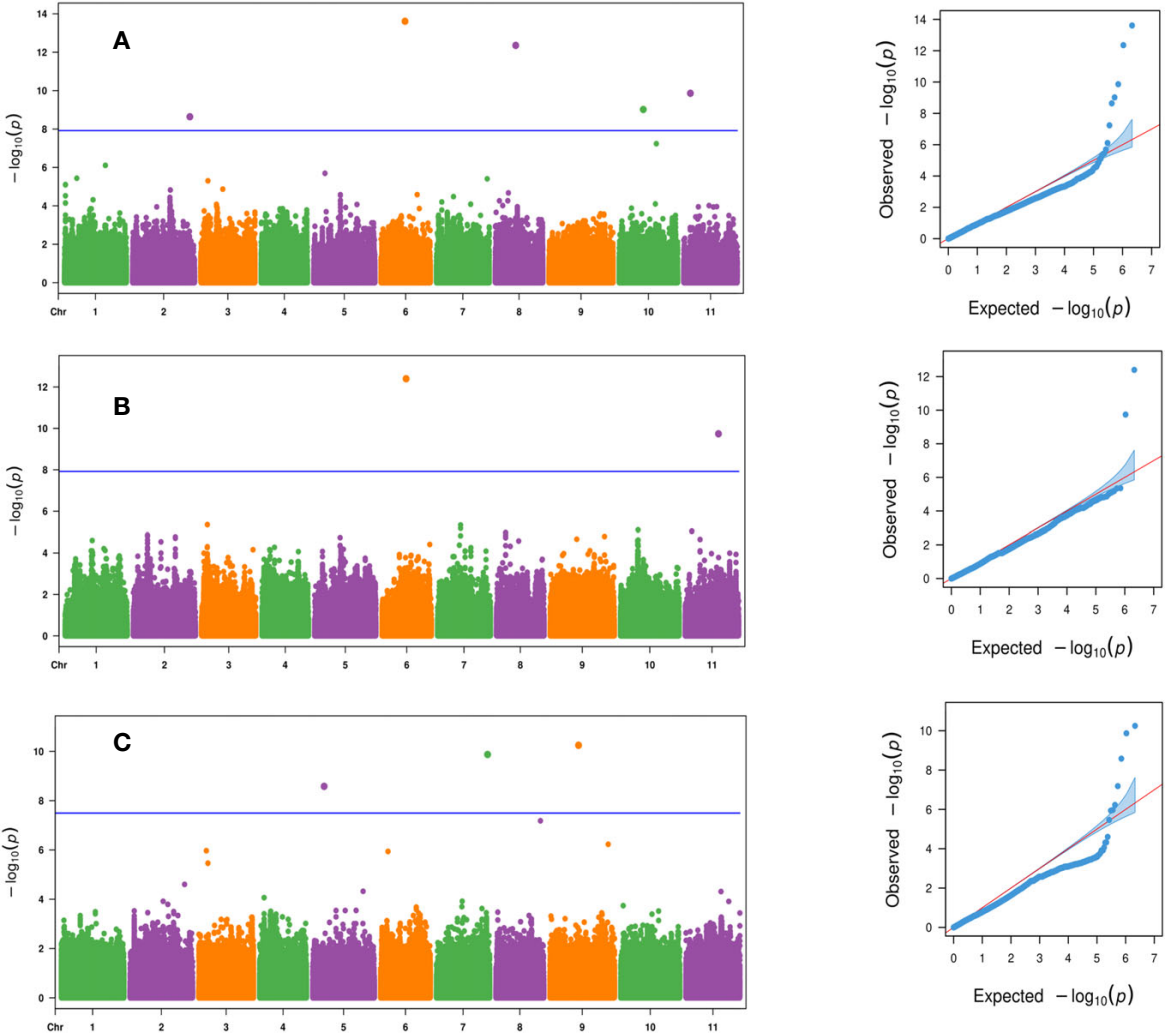
Manhattan and QQ plots for the evaluated traits of **(A)** fruit weight based on the FarmCPU model, **(B)** fruit weight based on the BLINK model, and **(C)** fruit yield based on the FarmCPU model across the 125 citron watermelon genotypes using BLUEs from the two field growing seasons. The blue solid horizontal line is an FDR cutoff of α = 0.05. QQ, quantile–quantile; BLUEs, best linear unbiased estimates; FDR, false discovery rate.

There were six significant SNPs associated with fruit weight (*p*- value ≤ 2.3 × 10^−9^) and were located on chromosomes Ca02, Ca06, Ca08, Ca10, and Ca11 (**Table 3**; **Figures 5A, B**). There was one significant SNP marker on each of chromosomes Ca02, Ca06, Ca08, and Ca10, while Ca11 had two SNPs, S11_3991224 and S11_20119275 (**Table 3**; **Figures 5A, B**). Four of the significant SNPs were uncovered only by the FarmCPU model, and one SNP was unique to BLINK. The most significant SNP on chromosome Ca06 (*p*- value = 2.5 × 10^−14^) was uncovered by both the BLINK and FarmCPU models (**Table 3**; **Figures 5A, B**). The phenotypic variation explained for fruit weight by these six significant SNP markers ranged from 1.6% to 25.4% (**Table 3**). Three significant SNPs were associated with fruit yield (*p*- value ≤ 2.6 × 10^−9^) and were distributed on chromosomes Ca05, Ca07, and Ca09 (**Table 3** and **Figure 5C**). All three significant SNP markers were uncovered using the FarmCPU model and explained 11.4% to 21.6% of the phenotypic variation in fruit yield (**Table 3** and **Figure 5C**).

Candidate genes relevant to flowering time, plant growth, development, and maturation were detected. For flowering time, three of the significant SNP markers co-located with five genes that have been reported to control time to flower across several crop systems. Candidate gene *CaU04G13940* was located at 79.4 kb downstream of SNP marker S4_27578802 on chromosome Ca04 and codes for a TCP transcription factor. On chromosome Ca05, SNP marker S5_34377129 co-located with two candidate genes. Candidate gene *CaU05G29300* was located at 72.8 kb upstream of SNP S5_34377129 and codes for WRKY transcription factor. Candidate gene *CaU05G29490* was found at 47.8 kb downstream of marker SNP S5_34377129 and codes for NAC domain protein. On chromosome Ca09, candidate gene *CaU09G17620* was located at 91.5 kb upstream of marker S9_27213639 and codes for a MADS-Box transcription factor. Candidate gene *CaU09G17740* was located at 45.7 kb downstream of SNP marker S9_27213639 and codes for a RING/U-Box protein.

For fruit count, candidate gene *CaU05G12790* was located at 70.7 kb downstream of SNP marker S5_10364285 on chromosome Ca05 and codes for NAC domain protein. For fruit weight, candidate gene *CaU02G24820* was located at 86.3 kb upstream of SNP marker S2_34729922 on chromosome Ca02 and codes for a MYB transcription factor. Candidate gene *CaU06G10680* was found at 5.5 kb downstream of marker S6_14558842 on chromosome Ca06 and codes for ethylene responsive transcription factor. Candidate gene *CaU11G04780* was located at 45.3 kb upstream of SNP S11_3991224 on chromosome Ca11 and codes for the embryonic flower-1 protein. Candidate gene *CaU11G04840* was found at 15.9 kb downstream of SNP S11_3991224 on chromosome Ca11 and codes for ethylene responsive transcription factor. For fruit yield, candidate gene *CaU05G09240* was located at 83.4 kb upstream of SNP S5_6875362 on chromosome Ca05 and codes for WD40 repeat protein. Candidate gene *CaU05G09240* was located at 2.9 kb downstream of marker S5_6875362 on chromosome Ca05 and codes for a phytochrome B (*PhyB*) photoreceptor. Candidate gene *CaU05G09420* was found at 53.5 kb downstream of SNP S5_6875362 on chromosome Ca05 and codes for a calmodulin protein. Candidate gene *CaU09G15300* was located at 51.9 kb downstream of the SNP marker S9_17168406 on chromosome Ca09 and codes for a MYB transcription factor.

### Allelic effects of the significant SNPs on the evaluated traits

**Figure 6:**
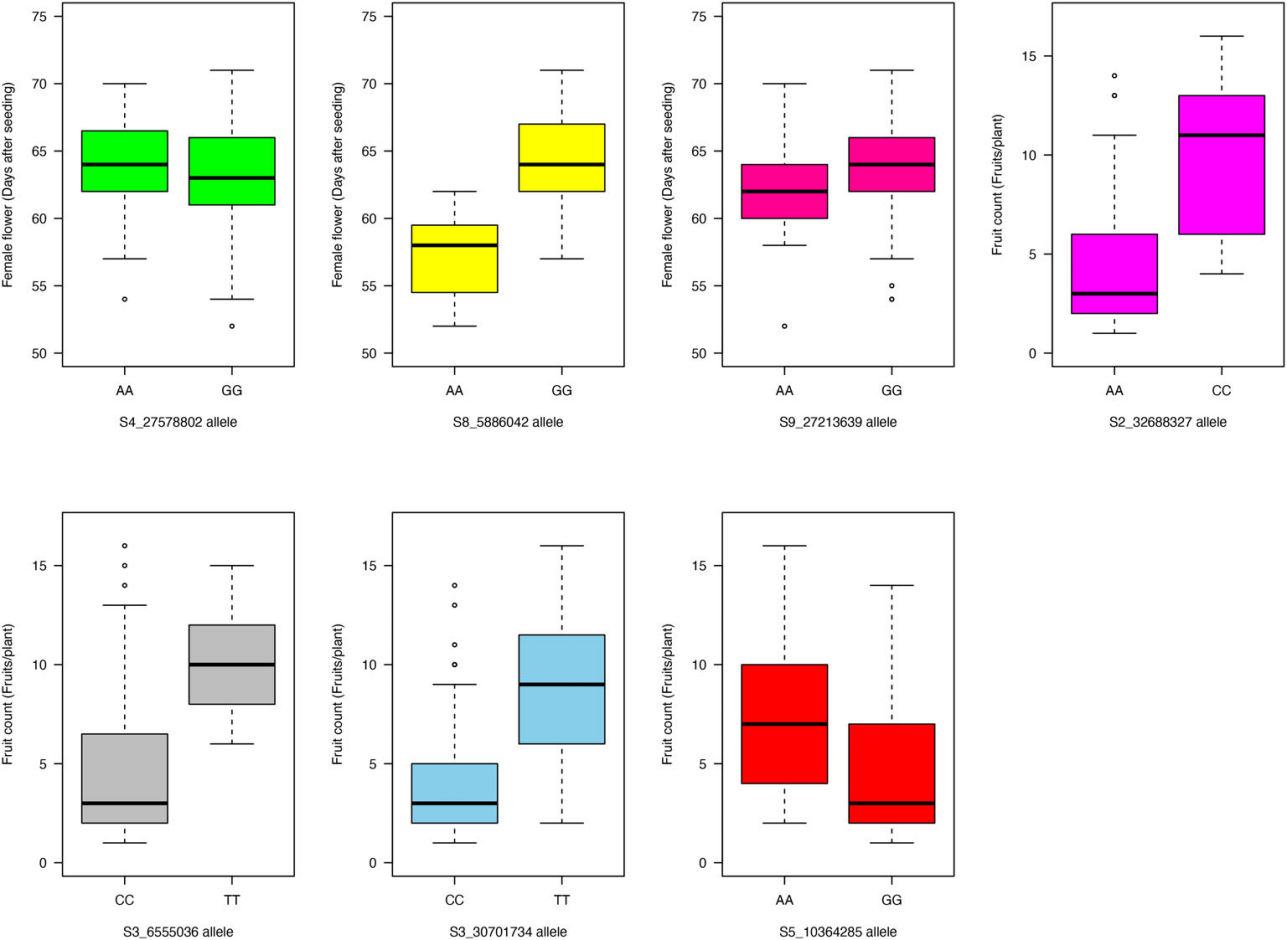
Boxplot showing allelic effects of the significant SNP markers associated with days to female flower and fruit count traits in the 125 citron watermelon genotypes based on BLINK and FarmCPU GWAS models. SNP, single-nucleotide polymorphism; GWAS, genome-wide association study.

**Figure 7:**
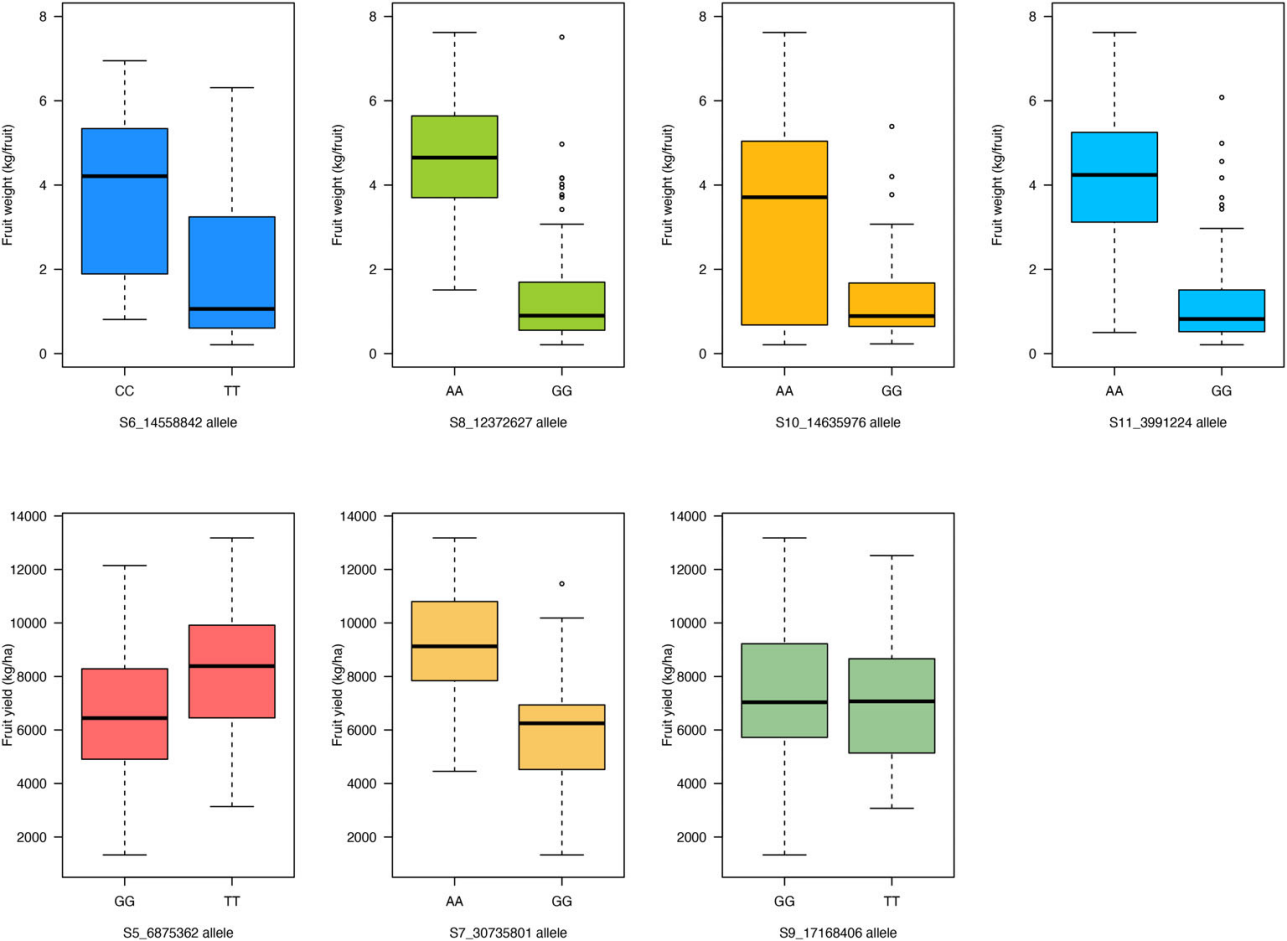
Boxplot showing allelic effects of the significant SNP markers associated with fruit weight and fruit yield traits in the 125 citron watermelon genotypes based on BLINK and FarmCPU GWAS models. SNP, single-nucleotide polymorphism; GWAS, genome-wide association study.

Allelic effects of the significant SNPs on the evaluated traits were explored (**Figures 6**, **7**). For the “AA” and “GG” alleles of SNP S4_27578802 on chromosome Ca04, there were no significant differences in days to female flower (**Figure 6**). The “AA” allele of SNP marker S8_5886042 on chromosome Ca08 was responsible for earlier female flowering (mean = 57 days after seeding) compared with a mean of 64 days after seeding among genotypes homozygous for the “GG” allele (*p*- value = 1.5 × 10^−5^; **Figure 6**). There was no significant difference between the “AA” and “GG” allelic classes of SNP marker S9_27213639 on days to female flower (**Figure 6**). For fruit count, genotypes with the “AA” allele for SNP S2_32688327 on chromosome Ca02 had fewer fruits per plant (mean = 4) compared with the “CC” allele (mean = 10 fruits per plant; *p*- value = 1.3 × 10^−4^) (**Figure 6**). The “CC” allele of SNP marker S3_6555036 resulted in a lower fruit count (mean = 5 fruits per plant) compared to the “TT” allele with a mean count of 10 fruits per plant at a *p*- value of 5.5 × 10^−4^ (**Figure 6**). Genotypes with the “CC” allele for SNP marker S3_30701734 on chromosome Ca03 had fewer fruits per plant (mean = 4) compared to the “TT” allele (mean = 9; *p*- value = 9.3 × 10^−7^) (**Figure 6**). For SNP S5_10364285, genotypes with the “AA” allele had a slightly higher fruit count average of seven fruits per plant compared to those with the alternative homozygous allele of “GG” (mean = 5 fruits per plant; *p*- value = 0.03) (**Figure 6**).

For fruit weight, the “CC” allele of SNP S6_14558842 on chromosome Ca06 had larger fruits (mean = 3.9 kg/fruit) compared to the “TT” allele (mean = 2 kg/fruit; *p*- value = 6 × 10^−6^) (**Figure 7**). Genotypes with the “AA” allele of SNP S8_12372627 had larger fruits (mean 4.6 kg/fruit) compared to the “GG” allele (mean = 1.4 kg/fruit; *p*- value = 7 × 10^−21^) (**Figure 7**). The “AA” allele of SNP S10_14635976 had a higher average fruit weight of 3.3 kg/fruit compared to the “GG” allelic class (mean = 1.4 kg/fruit; *p*- value = 2.6 × 10^−7^) (**Figure 7**). For SNP marker S11_3991224 on chromosome Ca11, the “AA” allele resulted in larger fruits (mean = 4.3 kg/fruit) compared to the “GG” allele (mean = 1.3 kg/fruit; *p*- value = 2.2 × 10^−17^) (**Figure 7**). For fruit yield, the “GG” allele of SNP S5_6875362 on chromosome Ca05 resulted in a lower mean fruit yield of 6,568.2 kg/ha compared to the “TT” allele (mean = 8,258.8 kg/ha; *p*- value = 2.6 × 10^−4^) (**Figure 7**). Genotypes with the “AA” allele for SNP S7_30735801 on chromosome Ca07 had a higher fruit yield (mean = 9,267.4 kg/fruit) compared to the “GG” allelic class (mean = 5,880.1 kg/ha; *p*- value = 1.9 × 10^−14^) (**Figure 7**). For SNP S9_17168406, the fruit yield performance of genotypes carrying either allelic class (“GG” or “TT”) was statistically similar (**Figure 7**).

## Discussion

Flowering time and fruit yield are crucial traits in watermelon breeding and cultivar development (Wehner, 2008). Flowering time can dictate crop maturity dates, escape from biotic and abiotic stressors, yield performance, and when the watermelon produce can be introduced to the market, ultimately influencing the produce prices. In this research, male flowers appeared earlier compared to female flowers as has been previously reported (Wehner, 2008; McGregor et al., 2014). Both flower types had moderately high heritability, suggesting the presence of a strong genetic component to the inheritance of these traits. Previous studies have detected flowering time QTL on chromosomes 3 and 11 (days to female flower) and chromosomes 2 and 3 (days to male flower) when working with biparental populations. This research revealed new genomic regions on chromosomes Ca04, Ca05, Ca08, and Ca09 controlling flowering time (days to female flower) in watermelon.

Fruit yield is a complex trait controlled by many loci and environmental factors. Detection of molecular markers associated with yield can help accelerate marker-assisted breeding. Fruit yield had a moderate heritability estimate, while yield component traits (fruit count and fruit weight) had moderately high heritability. These results are similar to earlier findings (Kumar and Wehner, 2011; Kumar and Wehner, 2013). A combined total of 16 SNP markers associated with fruit yield and fruit yield component traits were detected in this research, suggesting the quantitative inheritance nature of these traits. Analysis of allelic effects of the significant SNPs showed the beneficial contribution of certain allelic states for days to female flower, fruit count, fruit weight, and fruit yield traits evaluated in this research. The germplasm lines carrying these beneficial allelic states of the significant SNP markers can be used in crossing blocks to improve watermelon for early flowering time and fruit yield component traits. Prior to this study, there have been limited research efforts to dissect the genetic architecture of yield and yield components in watermelon. The molecular marker information generated in this research should provide a foundation for marker-assisted breeding of fruit yield in watermelon.

Trait correlation analysis was explored to help explain relationships among flowering time and yield component traits. Both days to male and female flowers were positively correlated, suggesting that it is feasible to select both phenotypes in a watermelon breeding program. Synchrony of male and female flowering times is very important, especially during the development of diploid pollenizers for triploid (seedless) watermelon breeding and production (Maynard and Elmstrom, 1992). Fruit count was negatively correlated with both fruit weight and fruit yield, implying a reduction in yield from many fruits per plant (heavy crop load). This is probably due to competition for photosynthetic assimilates that have to be partitioned across a large number of fruits, consequently affecting the overall yield per hectare. Fruit weight was positively correlated with fruit yield, suggesting that the two traits can be improved concurrently. Average fruit weight is a crucial trait and key determinant of marketable and non-marketable portions of the watermelon produce, as strict fruit size grades need to be met before delivery to markets (Kaiser, 2012).

Five candidate genes related to flowering time were detected in this research. Initiation of flowering in plants is a complex genetic process that requires both environmental cues (temperature and heat) and internal/developmental factors such as hormonal status, transcription factors, sugars, and age-dependent signals (Corbesier et al., 1998; Blazquez, 2000; Legris et al., 2016; Ramya et al., 2017; Zhang et al., 2018). Three of the candidate genes were transcription factors (TCP, WRKY, and MADS-Box), while the remaining two genes were NAC domain and RING/U-Box proteins. The TCP, WRKY, and MADS-Box transcription factors have been reported to regulate seed germination, vegetative growth, flower formation, and fruit development across many crop systems including tomatoes and *Arabidopsis* (Borner et al., 2000; Kaufmann et al., 2009; Balsemao-Pires et al., 2013; Luo et al., 2013; Zhang et al., 2018). Both NAC domain and RING/U-Box proteins have been reported to regulate time to flower and tolerance to abiotic stress in *Arabidopsis* (Kim et al., 2007; Yoo et al., 2007; Zhou et al., 2015). Seven candidate genes were located in close proximity to the significant SNP markers associated with fruit yield component traits evaluated in this research. Overall, these fruit yield-related candidate genes have previously been reported to be involved in plant growth and development, flowering time, light sensing, and fruit weight and size. For instance, WD40 repeat proteins play important roles in many plant development processes including cell division, floral development, and meristem organization (Stirnimann et al., 2010). Phytochrome B (*PhyB*) is a crucial photoreceptor that orchestrates multiple signaling pathways in plants to ensure optimal growth and development and transition to flowering (Legris et al., 2016). These candidate genes warrant further investigation and analysis to determine their functional mechanisms and develop markers for early flowering and fruit yield improvement in watermelons.

## Conclusion

This research characterized variation for flowering time (days to male and female flowers), fruit yield, and fruit yield component traits. Moderate-to- high heritability estimates of these traits have been generated. Twenty SNP markers and several candidate genes associated with the evaluated traits across the citron watermelon chromosomes have been identified. These genomic regions and marker effects can help guide genomics-assisted breeding for phenological and fruit yield component traits in watermelon.

## Data availability statement

The data presented in this study can be found in online repositories at the following cucurbit genomics websites: (http://cucurbitgenomics.org/; http://cucurbitgenomics.org/ftp/genome/watermelon/USVL246/; http://cucurbitgenomics.org/ftp/reseq/watermelon/Amarus_reseq/).

## Author contributions

DNK designed and implemented the project, performed the experiments, collected and analyzed the data, visualized and interpreted the results, and wrote the manuscript. AL designed and implemented the project. WW designed and implemented the project. All authors contributed to the article and approved the submitted version.
